# Bed demand and occupancy within the Brazilian National Health System for the most common types of cancer in Brazil, from 2018 to 2021: a cross-sectional study

**DOI:** 10.1590/S2237-96222024v33e20231172.en

**Published:** 2024-08-23

**Authors:** Mariana Araujo Neves Lima, Daniel Antunes Maciel Villela

**Affiliations:** 1 Fundação Oswaldo Cruz, Escola Nacional de Saúde Pública Sergio Arouca, Rio de Janeiro, RJ, Brazil; 2 Fundação Oswaldo Cruz, Programa de Computação Científica da Fiocruz (Procc), Rio de Janeiro, RJ, Brazil; 3 Center for Health Wellbeing, School of Public and International Affairs, Princeton University, Princeton, United States of America

**Keywords:** Acceso a Servicios de Salud, Equidad en el Acceso a los Servicios de Salud, Neoplasias, Investigación operativa, Teoría de colas, Access to Health Services, Equity in Access to Health Services, Neoplasms, Operational Research, Queuing Theory

## Abstract

**Objective:**

To analyze bed demand and occupancy within the Brazilian National Health System (*Sistema Único de Saúde* – SUS) for the main types of cancer in Brazil, from 2018 to 2021.

**Methods:**

This was a descriptive cross-sectional study, using data from the Hospital Information System. Queuing theory model was used for calculating average admission rate, average hospitalization rate, probability of overload, and average number of people in the queue.

**Results:**

The Southeast and South regions showed the highest average hospitalization rates, while the North region showed the lowest rates. The Southeast region presented a high probability of surgical bed overload, especially in the states of São Paulo (99.0%), Minas Gerais (97.0%) and Rio de Janeiro (97.0%). São Paulo state showed an overload above 95.0% in all types of beds analyzed.

**Conclusion:**

There was a high probability of oncology bed occupancy within the Brazilian National Health System, especially surgical and medical beds, and regional disparities in bed overload.

## Study contributions

**Main results:**

The study found a high demand for hospital admissions to oncological bed in the Southeast region and a high probability of system overload in the states of the Southeast and Northeast regions of Brazil, thus highlighting the inequities in access to healthcare services in the country.

**Implications for services:**

This study presents a methodology for the improved allocation of resources and management of surgical and medical bed flows in areas with the highest bed overload and regions with low service availability.

**Perspectives:**

It is necessary to promote public policies that ensure the equitable supply of beds for oncological treatment within the SUS, especially in states with bed overload and healthcare service gaps.

## RESUMEN

**Objetivo:**

Evaluar la demanda y ocupación de camas en el Sistema Único de Salud (SUS) de los principales tipos de cáncer en Brasil, de 2018 a 2021.

**Métodos:**

Estudio descriptivo transversal, utilizando datos del Sistema de Información Hospitalaria. Se realizaron cálculos basados en el modelo de teoría de colas de: tasa promedio de ingreso por hospitalización, tasa promedio de hospitalización, probabilidad de sobrecarga y número promedio de personas en lista de espera.

**Resultados:**

Las regiones Sur y Sureste presentaron las tasas promedio de hospitalización más altas, mientras que la región Norte tuvo las tasas más bajas. La región Sureste presentó alta probabilidad de sobrecarga de camas quirúrgicas, sobre todo San Pablo (99,0%), Minas Gerais (97,0%) y Río de Janeiro (97,0%). San Pablo presentó sobrecarga superior al 95,0% en todos los tipos de camas analizadas.

**Conclusión:**

Hubo alta probabilidad de ocupación de camas de oncología en el SUS, mayormente en quirúrgicas y clínicas, además de disparidades regionales en la sobrecarga de camas.

## INTRODUCTION

Brazil shows an upward trend in the incidence of cancer due to population aging, lifestyle changes, and increased environmental and occupational exposures^1^. According to the most recent data on cancer incidence in Brazil, over 700 thousand new cases of cancer were estimated in 2023.^1^ Among them, the most common were breast, prostate, colorectal, lung, stomach, and cervical.^1^

Brazilian National Health System consists of a regionalized and hierarchical network aimed to provide comprehensive and universal care for the Brazilian population^2^. The structuring of Health Care Network encompasses everything from health promotion, cancer prevention and screening to palliative care. The hospital oncology care network is comprised of High-Complexity Oncology Care Units (*Unidades de Assistência de Alta Complexidade em Oncologia* – UNACON) and High Complexity Oncology Care Centers (*Centros de Assistência de Alta Complexidade em Oncologia* – CACON).^3^

Although there is a hospital oncology care network, previous studies have found regional inequalities in access to health services within the Brazilian National Health System. These studies sought to identify the origin-destination flow of hospitalizations among people with breast cancer,^4, 5^ digestive system cancer^6^ and for types of treatment such as chemotherapy, radiotherapy and surgery.^7^ The Southeast and Northeast regions are hubs for oncological care, that is, they attract a higher demand for care, which may lead to queues in these regions, and healthcare service gaps, especially in the North region.^4,5,^ 7

In Brazil, there is a shortage of general hospital beds, especially within the Brazilian National Health System. In 2017, countries in the Organization for Economic Co-operation and Development (OECD) had an average of 4.7 hospital beds per 1,000 people, while in Brazil the average was 2.3 beds per 1,000 inhabitants.^8^ Regarding ICU beds, in 2020, the country had 1.4 beds within the Brazilian National Health System per 10,000 inhabitants, compared to 4.9 in the private sector.^9^ In addition, states such as Amapá, Roraima, Acre, Maranhão, Piauí and Bahia have fewer than 1 bed per 10,000 inhabitants, which is fewer than the number recommended by the World Health Organization of 1 to 3 beds per 10,000 inhabitants.^10^

Although there is no recommended number of beds, the Ministry of Health proposed criteria and parameters for determining the number of general beds taking into account four components.^11^ A comparative study on the supply parameters between the ordinances of 2002 and 2015 found that the distribution of general and ICU bed supply across the country is not ideal to serve the entire population equitably.^12^ The unavailability of beds for cancer treatment is critical, as users usually require surgical beds for curative or palliative surgery, medical beds for hospitalizations, treatment of intercurrences and complications, and Intensive Care Unit (ICU) beds. In cases of insufficient beds, there may be a cancellation of elective surgeries, the failure in the transfer flow between care units, and inappropriate use of beds, which is also related to bed management.^1^ Therefore, it can lead to delays in starting treatment.

Thus, assessing system overload helps health managers in decision-making regarding the planning of the ideal health service capacity, improvements in care flows for users with cancer and in resource allocation.^14^ Therefore, this study used the queuing theory mode^15^ with the objective of analyzing bed demand and occupancy within the Brazilian National Health System for the most common types of cancer in Brazil, from 2018 to 2021.

## METHODS

### Study design

This was a descriptive cross-sectional study, using data on hospitalizations for acute and chronic medical conditions directly related to cancer, reported between 2018 and 2021.

### Setting

The hospital network for oncological care in Brazil comprises 359 registered institutions. It is worth mentioning that 265 hospitals are accredited as High-Complexity Oncology Care Units (*Unidades de Assistência de Alta Complexidade em Oncologia* -UNACON), where the most common types of cancer in the country are treated, and 44 units are High-Complexity Oncology Care Centers (*Centros de Assistência de Alta Complexidade em Oncologia* – CACON) providing care for all types of cancer^16^. In 2019, Ordinance GM/MS No. 139^9^ 17 established the presence of one accredited hospital per 1,000 new cancer cases estimated annually.

Between 2018 and 2021, 898,724 hospitalizations were performed, of which 224,532 in 2018, 239,603 in 2019, 220,559 in 2020 and 214,030 in 2021, resulting in an average of 2019.5 hospitalizations per year. In this study, records with hospitalization date and discharge date between 1/1/2018 and 12/31/2021 were selected, according to Federative Unit (FU). This analysis period was considered based on the availability of updated data. As it covered two years of the COVID-19 pandemic, we decided to include the two years preceding them.

### Participants

The study sample comprised hospitalizations registered in the Hospital Information System (*Sistema de Informação Hospitalar do Sistema Único de Saúde*, SIH/SUS), between 01/01/2018 and 12/31/2021, with the following principal diagnosis codes from the International Statistical Classification of Diseases and Related Health Problems 10^th^ Revision (ICD-10): C50 (breast cancer), C53 (cervical cancer), C61 (prostate cancer), C34 (bronchial and lung cancer) and codes C18 to C20 (colorectal cancer).

### Variables

- Hospital Admission Authorization number (HAA);
- Primary diagnosis codes (ICD-10): breast cancer (ICD-10: C50), cervical cancer (ICD-10: C53), prostate cancer (ICD-10: C61), bronchial and lung cancer (ICD-10: C34), and colorectal cancer (ICD-10: C18 to C20)
- Hospitalization date;
- Discharge date;
- Sex: female and male;
- Age group in years: up to 18, 19 to 39, 40 to 59, 60 to 69, 70 to 79, 80 to 89, 90 and older;
- Race/skin color: asian, white, indigenous, mixed-race and black;
- Region: Midwest, Northeast, North, Southeast, South;
- Year of treatment: 2018, 2019, 2020 and 2021;
- Type of bed: surgical, medical, and others;
- Hospitalization for acute or chronic conditions and treatment of intercurrences and complications;
- Hospitalization for sequential surgical procedures: consists of hospitalization in which the surgical procedures occur under the same anesthetic procedure;
- Hospitalization for continuous chemotherapy treatment: hospitalization for 24-hour continuous intravenous infusion chemotherapy.

### Queuing theory model measures

- Average admission rate (λ): corresponds to the average rate of users arriving to be treated in a hospital bed per day. This variable represents the demand for hospitalization;
- Average hospitalization rate (μ): consists of the number of hospitalizations per unit of time, which is the variable representing the supply of services in the system;
- Probability of overload (ρ): refers to the congestion of services. This measure is calculated by the equation $P(n>0)=\rho =\left(\frac{\lambda }{µ}\right)$

- Average number of users in the queue (Lq): queue Length, which represents the average number of users in the queue waiting for a hospital bed. It is calculated using the following equation: Lq = 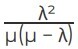 ^15^

### Data source

Data on hospital admissions to medical and surgical beds and in the Intensive Care Unit (ICU) were collected through the Hospital Information System (*Sistema de Informação Hospitalar do Sistema Único de Saúde* – SIH-RD/SUS).^18^ As such, when collecting data on hospital admissions during the analysis period, the same user may have undergone several hospitalizations. Therefore, each hospitalization in a hospital bed was the result of a queue for that bed. For automatic data extraction, the microdatasus package available on the R platform, was used.^19^

### Statistical analysis

Queuing theory was used for the analysis, in which the FUs were analyzed as admission units for users with symptoms and with the capacity to provide diagnostic and treatment services. The main components of the queue model include: average arrival rate (λ), average service rate (μ), number of servers, service capacity, and queue discipline, i.e., the order in which users arrive.

Initially, a descriptive analysis of the characteristics of the study population (sex, age group, race/skin color, region, year, and type) was performed. The average arrival rate was defined as the average admission rate and the service rate as the average hospitalization rate.

As these were hospital admissions to hospital beds, the average length of hospital stay was first calculated (Supplementary Table 1). In this indicator, the numerator was the sum of the number of users per day in the study period and the denominator was the number of discharges during the same period. In order to calculate the number of users per day, a database was constructed, in which each arrival corresponded to an individual hospitalization.

**Table 1:** Distribution of hospitalizations for the most common types of cancer in Brazil according to sociodemographic variables and types of bed, 2018-2021

**Variables**	**Total** **N = 898,724**	**Cervical** **cancer** **N = 90,039**	**Colorectal cancer** **N = 314,537**	**Breast cancer** **N = 271,861**	**Prostate cancer** **N = 125,519**	**Lung cancer** **N = 96,768**
	**n (%)**	**n (%)**	**n (%)**	**n (%)**	**n (%)**	**n (%)**
**Sex**						
Female	55,054 (62.0)	90,021 (100.0)	153,633 (49.0)	268,988 (99.0)	NA	44,412 (46.0)
Male	341,670 (38.0)	NA	160,904 (51.0)	2,873 (1.0)	125,519 (100.0)	52,356 (54.0)
**Age group (in years)**						
Up to18	4,532 (0.5)	105 (0.1)	2,830 (0.9)	796 (0.3)	170 (0.1)	631 (0.7)
19 to 39	82,091 (9.1)	26.235 (29,0)	21.185 (6,7,0)	31.365 (12,0)	177 (0,1)	3,129 (3.2)
40 to 59	342,546 (38.0)	43,001 (48.0)	116,281 (37.0)	137,641 (51.0)	18,525 (15.0)	27,098 (28.0)
60 to 69	251,755 (28.0)	12,536 (14.0)	94,500 (30.0)	60,843 (22.0)	48,416 (39.0)	35,460 (37.0)
70 to 79	161,479 (18.0)	6,251 (6.9)	60,379 (19.0)	30,463 (11.0)	41,165 (33.0)	23,221 (24.0)
80 to 89	51,012 (5.7)	1,738 (1.9)	17,889 (5.7)	9,631 (3.5)	15,058 (12.0)	6,696 (6.9)
90 and older	5,309 (0.6)	173 (0.2)	1,473 (0.5)	1,122 (0.4)	2,008 (1.6)	533 (0.6)
**Race/skin/color**						
Asian	12,068 (1.5)	1,498 (1.9)	4,065 (1.4)	3,442 (1.4)	1,674 (1.6)	1,389 (1.7)
White	414,577 (52.0)	33,133 (43.0)	170,413 (61.0)	118,966 (49.0)	46,104 (43.0)	45,961 (56.0)
Indigenous	343 (< 0.1)	135 (0.2)	85 (< 0.1)	61 (< 0.1)	28 (< 0.1)	34 (< 0.1)
Mixed-race	314,118 (40.0)	38,413 (49.0)	93,565 (33.0)	103,272 (42.0)	49,053 (46.0)	29,815 (36.0)
Black	50,203 (6.3)	4,775 (6.1)	12,705 (4.5)	17,385 (7.2)	10,705 (10.0)	4,633 (5.7)
**Region**						
Midwest	51,712 (5.8)	6,393 (7.1)	17,670 (5.6)	14,945 (5.5)	7,050 (5.6)	5,654 (5.8)
Northeast	177,630 (20.0)	23,409 (26.0)	44,137 (14.0)	60,970 (22.0)	30,947 (25.0)	18,167 (19.0)
North	28,846 (3.2)	7,048 (7.8)	6,265 (2.0)	8,773 (3.2)	3,597 (2.9)	3,163 (3.3)
Southeast	425,812 (47.0)	35,850 (40.0)	144,538 (46.0)	136,769 (50.0)	64,889 (52.0)	43,766 (45.0)
South	214,724 (24.0)	17,339 (19.0)	101,927 (32.0)	50,404 (19.0)	19,036 (15.0)	26,018 (27.0)
**Year of treatment**						
2018	222,479 (25.0)	21,876 (24.0)	76,289 (24.0)	67,994 (25.0)	32,394 (26.0)	23,926 (25.0)
2019	239,360 (27.0)	23,948 (27.0)	81,506 (26.0)	73,093 (27.0)	34,714 (28.0)	26,099 (27.0)
2020	221,196 (25.0)	22,438 (25.0)	78,813 (25.0)	65,884 (24.0)	29,582 (24.0)	24,479 (25.0)
2021	215,689 (24.0)	21,777 (24.0)	77,929 (25.0)	64,890 (24.0)	28,829 (23.0)	22,264 (23.0)
**Types of bed**						
Surgical	426,081 (47.0)	39,088 (43,0)	118,919 (38.0)	175,940 (65.0)	71,630 (57.0)	20,504 (21,0)
Medical	449,465 (50.0)	47.,310 (53.0)	189,781 (60.0)	89,771 (33.0)	50,167 (40.0)	72,436 (75.0)
Others	23,178 (2.6)	3,641 (4.0)	5,837 (1.9)	6,150 (2.3)	3,722 (3.0)	3,828 (4.0)

NA: Not applicable.

The modeling of the average admission rate was performed using the Poisson process with exponential distribution, since it assumes a discrete distribution of events with a large number of users having independent admissions.^15^ In this case, the events are the new hospital records per unit of time (day). This assumption was evaluated graphically.

The M/M/1 model was used, given that the intervals between arrivals and the average hospitalization rates follow an independent and identically distributed exponential distribution. In this model, M stands for Markovian and 1 describes a queue with a single server which serves users in the order in which they arrive. This model is characterized by the first-in-first out (FIFO) service discipline. In this study, the queue results from a single server for hospital beds and the service is provided in the order of arrival, as users are referred to the treatment unit via the SUS referral system.

Based on the parameters of average admission rate (λ) and average hospitalization rate (μ), the probability of overload (ρ) and the average number of users in the queue (Lq) can be calculated. When the average admission rate (λ) is higher than the discharge rate (μ), it indicates an increase in the probability of queues.

### Ethical considerations

This study used secondary, non-identifiable data, therefore it was exempted from registration and approval of a Research Ethics Committee.

## RESULTS

A total of 912,567 hospitalizations for the types of cancer analyzed between 2018 and 2021 were selected. After excluding incomplete data, there were 898,724 records of HAA for people with breast cancer (N = 271,861), cervical cancer (N = 90,039), colorectal cancer (N = 314,537), prostate cancer (N = 125,519), and lung cancer (N = 96,768) (Table 1).

The study population was comprised mostly of females (62.0%) aged between 40 and 69 years (66.0%). Among hospitalizations for cervical cancer, the age group with the highest frequency was between 40 and 59 years (48.0%). Approximately 50% of hospitalizations for breast cancer were among women aged between 40 and 59 years, whereas hospitalizations for prostate cancer, 84.0% of cases were aged between 60 and 89 years. This age group was also predominant in lung cancer cases (68.0%). Regarding hospitalizations for colorectal cancer, the age group of 40 to 59 years (37.0%) stood out (Table 1).

The distribution of hospitalizations for colorectal cancer was similar between sexes, while 54% of hospitalizations for lung cancer were among male. The majority of records were from individuals of White race/skin color (52.0%). The Southeast region accounted for 47.0% of hospitalizations. For cervical cancer, the Southeast region presented 40.0% of hospitalizations and the Northeast region showed 26.0% of hospitalizations. Regarding colorectal cancer, the regions with the highest percentage of hospitalizations were Southeast (46.0%) and South (32.0%). The Southeast region accounted for 50.0% of hospitalizations for breast cancer and 52.0% for prostate cancer. With regard to lung cancer, 45.0% of hospitalizations occurred in the Southeast region and 27.0% in the South region. The highest number of hospitalizations was observed in 2019 (239,360 hospitalizations). Hospital admissions to medical beds were more frequent among people with colorectal cancer (60.0%) and lung cancer (75.0%); while hospitalizations for breast cancer (65.0%) and prostate cancer (57.0%), were more frequent in surgical beds. All variables were statistically significant (Table 1).

The Southeast and South regions showed high average hospitalization rates for surgery, especially in the states of São Paulo (525.0), Minas Gerais (269.9), Paraná (173.1) and Rio de Janeiro (169.9). On the other hand, the North region presented the lowest average hospitalization rates as it can be seen in the states of Acre (11.3), Amapá (12.0), Tocantins (15.7) and Roraima (16.4). The probability of overload was also high in the Southeast region, with 99.0% in São Paulo and 97.0% in both Minas Gerais and Rio de Janeiro. A total of 383 people per day waited in line for a surgical bed in the state of São Paulo (Table 2).

**Table 2:** Queue performance measures for hospitalizations for the most common types of cancer in Brazil according to medical, surgical and ICU beds, 2018-2021

**FU**	**Medical bed**				**Surgical bed**				**ICU bed**			
	**Average admission rate (**λ**)**	**Average hospitalization rate ( μ )**	**Probability of overload (**ρ**) %**	**Average number of users in the queue (Lq)**	**Average admission rate (**λ**)**	**Average hospitalization rate ( μ )**	**Probability of overload (**ρ**) %**	**Average number of users in the queue ( Lq)**	**Average admission rate (**λ**)**	**Average hospitalization rate ( μ )**	**Probabil ity of overload (**ρ**) %**	**Average number of users in the queue (Lq)**
São Paulo	473.1	478.2	99.0	437.1	517.8	525.0	99.0	383.5	104.3	110.0	95.0	93.9
Minas Gerais	269.1	274.2	98.0	234.1	261.2	269.9	97.0	154.9	54.2	60.6	89.0	40.2
Rio Grande do Sul	195.0	199.6	98.0	191.0	158.6	165.9	96.0	131.3	22.8	28.3	84.0	20.3
Paraná	286.8	294.6	97.0	155.3	163.9	173.1	95.0	92.0	34.9	43.4	80.0	17.7
Rio de Janeiro	153.8	158.1	97.0	171.5	164.1	169.9	97.0	149.4	33.2	37.7	88.0	38.0
Santa Catarina	119.0	124.4	95.0	91.9	91.7	100.3	93.0	58.1	14.5	20.9	74.0	8.8
Pernambuco	116.4	121.8	95.0	98.6	92.4	100.0	92.0	52.5	15.9	21.6	73.0	10.5
Bahia	79.0	82.9	95.0	96.6	115.9	124.0	93.0	65.7	17.2	22.6	76.0	13.0
Espírito Santo	75.3	81.2	92.0	53.3	56.0	64.8	87.0	28.7	12.3	19.3	66.0	6.1
Maranhão	36.6	40.9	90.0	40.0	39.2	46.6	85.0	23.2	12.2	17.5	71.0	8.5
Ceará	32.3	36.2	90.0	40.3	72.4	79.8	91.0	43.8	10.5	16.4	69.0	8.3
Distrito Federal	31.7	35.5	89.0	37.2	28.4	34.6	83.0	19.6	6.5	10.5	64.0	5.3
Pará	21.1	24.3	87.0	29.5	23.6	27.6	85.0	23.1	8.5	12.1	71.0	9.2
Rio Grande do Norte	43.9	50.1	86.0	26.0	39.0	48.4	82.0	17.9	9.5	17.3	56.0	3.7
Alagoas	36.6	42.1	86.0	26.1	21.9	30.3	74.0	10.0	6.9	13.2	52.0	2.9
Goiás	35.5	41.6	85.0	24.4	72.4	79.8	91.0	43.8	9.1	16.8	61.0	4.3
Paraíba	24.1	29.2	82.0	19.3	32.1	39.5	81.0	16.6	8.8	15.0	60.0	4.9
Mato Grosso do Sul	30.6	37.2	81.0	17.6	22.8	30.2	78.0	12.9	6.8	13.7	51.0	2.6
Piauí	17.9	22.1	81.0	16.8	27.1	35.0	78.0	13.0	6.9	12.9	54.0	3.6
Sergipe	9.3	11.5	81.0	17.0	12.8	19.9	69.0	7.0	5.8	9.6	63.0	5.5
Mato Grosso	25.2	31.1	80.0	15.5	23.5	30.9	78.0	12.7	8.0	14.7	58.0	3.7
Amazonas	11.3	14.9	78.0	12.7	17.8	23.7	77.0	11.7	6.3	11.1	59.0	4.1
Rondônia	18.3	24.3	75.0	11.5	14.4	22.0	68.0	6.6	7.2	14.6	51.0	2.7
Tocantins	14.8	19.6	75.0	11.6	10.2	15.7	69.0	7.4	5.5	10.4	53.0	3.2
Amapá	6.5	9.2	72.0	9.1	6.4	12.0	60.0	4.0	5.1	8.5	60.0	5.2
Acre	6.9	10.7	65.0	6.0	6.2	11.3	58.0	4.6	5.4	9.6	57.0	4.0
Roraima	7.5	12.0	64.0	5.4	6.4	16.4	43.0	1.6	5.1	10.3	50.0	2.7

FU: Federative Unit; ICU: Intensive Care Unit; λ: Average admission rate (hospitalized individuals/day); μ: Average hospitalization rate (hospitalized individuals/day); ρ: Probability of an overloaded system (%); Lq: Average number of users in the queue (individuals/day).

This study showed that the average daily hospital admissions to medical beds in the state of São Paulo was 473.1 people per day and 437.1 people per day waited to be seen in the state, resulting in 99.0% probability of system overload. Among the states with more than 95.0% probability of overload for medical beds, there were three in the Southeast region, three in the South region, and two in the Northeast region, specifically Bahia and Pernambuco (Table 2).

A total of 75,807 ICU beds were analyzed. When compared to other types of beds analyzed, ICU beds were less overloaded and, therefore, had fewer users waiting in line. However, the state of São Paulo still showed a 95% probability of ICU bed occupancy. The North and Northeast regions showed low arrival rate for ICU beds (Table 2).

**Figure 1:**
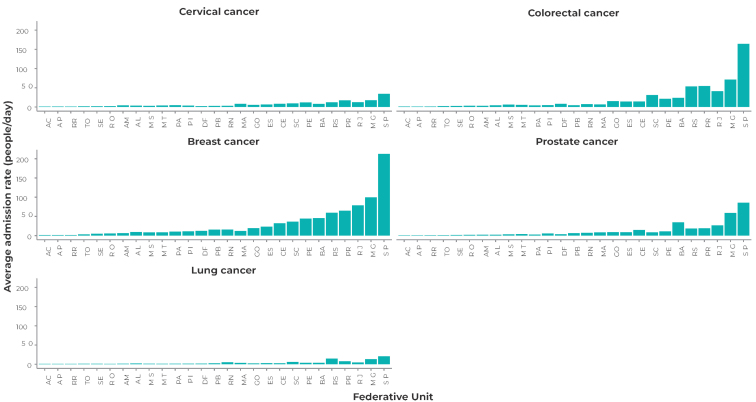
Average admission to surgical bed rate by Federative Unit and diagnosis, Brazil, 2018-2021

**Figure 2:**
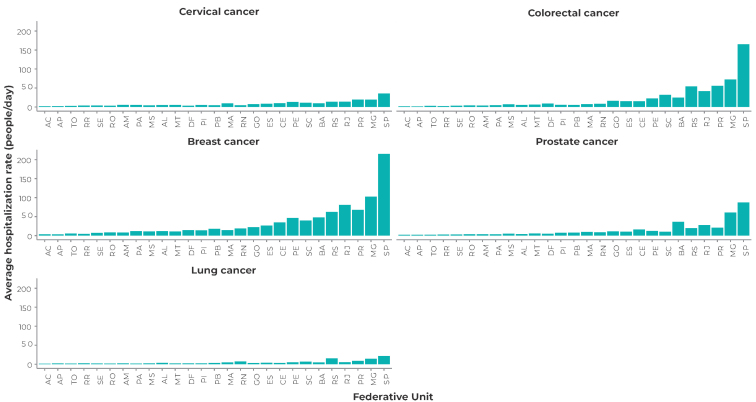
Average hospital admission to surgical bed rate by Federative Unit and diagnosis, Brazil, 2018-2021

**Figure 3:**
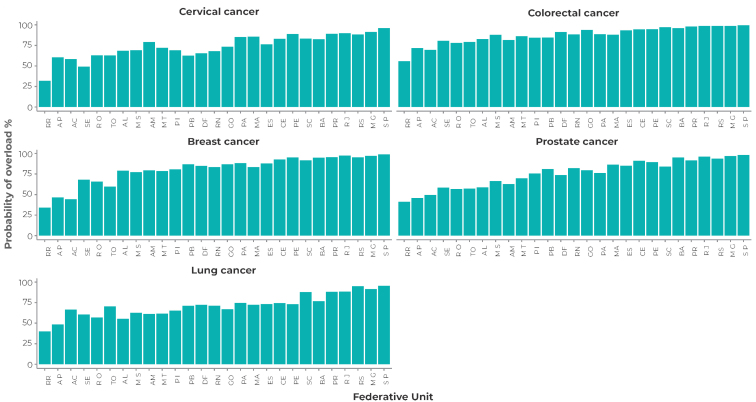
Probability of system overload for surgical beds by Federative Unit and diagnosis, 2018-2021

Figures 1 and 2 show that there was a higher average admission rate and a high average hospitalization rate in surgical beds for breast cancer and colorectal cancer. The state of São Paulo presented the highest admission rates, as well as the highest average hospitalization rates for all types of cancer analyzed. Hospitalizations for colorectal cancer showed the greatest probability of healthcare network overload, with the states of São Paulo, Rio de Janeiro, Minas Gerais and Rio Grande do Sul showing a 99.0% probability of surgical bed overload for this type of cancer. The probability of demand overload for surgical beds for breast cancer cases was also high, especially in the states of São Paulo (99.0%), Rio de Janeiro (97.0%) and Minas Gerais (97.0%) (Figure 3). Surgical procedures showed a high probability of bed occupancy and a high number of users in the queue (Supplementary Table 2, Supplementary Figure 1).

## DISCUSSION

The findings of this study corroborate those of other studies, which have found regional disparities in the supply of oncology beds in the public health network in Brazil^.4,5,^ 7 The states in the Southeast and South regions showed higher average hospitalization rates (μ) when compared to the North region, which presented lower hospitalization rates for surgical, medical and ICU beds. Regarding the types of cancer, colorectal showed a higher probability of surgical, medical, and ICU bed overload, with an estimated 654 users with colorectal waiting in line for surgical beds. Among the procedures analyzed, partial colectomy and abdominal recto-sigmoidectomy showed a 99% probability of bed overload.

The shortage of hospital beds within the SUS was also observed in a study that found a rate of 1.6 SUS beds per1,000 inhabitants in the North region and 5.0 and 5.2 private beds per 1,000 inhabitants in the North and Midwest regions, respectively.^20^ The findings of this study corroborate these estimates, as evidenced by the rates found in the states in the North region, which showed the lowest average hospitalization rates, highlighting the insufficient bed availability in the region, which calls for actions aimed at reducing these inequities.

The North region was considered the most critical region in a previous study,^21^ because it has only 12 accredited facilities for cancer treatment, including 11 high-complexity cancer services. Consequently, users from the North and Midwest regions experienced greater travel distance to treatment centers, however, the Southeast and Northeast regions are hubs, with the municipality of Barretos, state of São Paulo, being the main hub in Brazil.^5, 7^ In this study, it can be seen that the low admission rate in the system in the North region suggests that users seek care in other regions, as evidenced by the low bed overload in the region and greater overload in states in the Northeast and Southeast regions.

Queues formation occur if user demand exceeds the system’s capacity to provide services within the time frame.^22^ Consequently, delays in bed availability can lead to cancellation of elective surgeries, delays in clinical and emergency procedures, allocation of inappropriate beds, without taking into consideration sex and type of bed.^23^ Thus, shortage of bed can affect patient survival, quality of life and cancer-related mortality.

A population-based study estimated the global demand for cancer surgery in 183 countries between 2018 and 2040 and found that the number of cancer cases with an indication for surgery will increase by 52% in this period, totaling 5 million procedures. The greatest absolute increase in cancer cases with an indication for surgery will occur in upper-middle-income and lower-middle-income countries.^24^ With the increase in demand for cancer surgery, adequate planning for workforce and supplies is essential to provide surgical services.

Although it was not the focus of this research, the COVID-19 pandemic, which began in 2020, may influence the findings of the study. The need for social isolation may have an impact on reducing the average admission rate, as well as bed availability may have an impact on decreasing the average hospitalization rate. Previous studies have found a decrease in the number of days of hospital stay and a reduction in surgical procedure volumes.^25, 26^

The M/M/1 queueing model used was consistent with the needs of the study, as this model describes a queue with a single server which serves customers in the order in which they arrive. Although there are several service units, bed regulation by the SUS forms a single queue, thus considering only one server. In addition, for elective oncological surgeries, the queue for surgical beds is served by a single server in order of arrival at the hospital, according to the surgery schedule.

This study took into account the average length of stay in hospital for calculating the system performance measures. Therefore, it encompassed the clinical differences of the types of cancer analyzed that may influence system overload. It could be seen the differences between colorectal cancer and prostate cancer. The latter showed a lower average length of hospital stay, resulting in a low bed overload.

Present study has limitations. The SIH database does not include users of the supplementary health system, making it impossible to differentiate the probability of bed overload between SUS and the private sector. The SIH does not classify procedures according to clinical severity, nor does it include clinical variables, such as cancer staging, factors that can influence the entry into and exit from the queue system.^18^ The database records SUS users by hospitalizations, which does not allow for the individualization of the selected sample. This could introduce a bias. given that the same patient may be included multiple times in the study. However, it is expected that such cases represent a small portion of the studied sample.

A limitation of the data used is related to the possible access bias in diagnosis. The state of São Paulo showed the highest admission and hospitalization rates, as it has a large population and a high number of diagnoses, consequently increasing the average admission rate. However, states with limited diagnostic capabilities, may have more cases than those reported, but the admission rate will be lower. Regarding the M/M/1 model, its limitation is the lack of incorporation of demographic variables, such as the size or the average distance to the hospital, which can impact the access to the reference unit and, consequently, the queue for hospital beds. In addition, it is noteworthy that the statistically significant results of the descriptive analysis on sociodemographic variables and types of bed may be influenced by the large number of observations.

It can be concluded that there is evidence of regional inequalities in access to clinical, surgical and ICU admissions in Brazil. The study showed a shortage of beds for cancer treatment among SUS users in the North region and a high probability of overload in the Southeast and Northeast regions. Furthermore, the study showed that people with colorectal cancer, in particular, have a high likelihood of facing queues for hospitalization.

## Supplementary Table 1

**Table:** Average length of stay (in days) in a hospital bed for breast, prostate, colorectal, cervical and lung cancer in Brazil, 2018-2021

Variables	Medical clinic beds	Surgical beds	ICU beds
Region			
North	8.8	6.4	12.0
Southeast	6.6	5.2	10.5
Northeast	7.3	4.4	9.7
Midwest	6.3	4.8	10.7
South	5.3	5.0	10.0
Diagnosis			
Lung cancer	8.3	7.2	10.1
Cervical cancer	8.2	4.5	12.0
Colorectal cancer	4.8	8.3	11.0
Prostate cancer	7.4	4.5	8.3
Breast cancer	6.9	2.8	8.9
Type of bed			
Medical	6.4	-	12.2
Surgical	-	5.0	9.7
Year			
2018	6.7	5.2	10.9
2019	6.7	5.1	10.6
2020	6.2	4.9	10.1
2021	6.0	4.7	9.7

ICU: Intensive Care Unit Surgical beds

## Supplementary Table 2

**Table:** Queue performance measures for hospital admissions to surgical bed by procedure, 2018-2021

Procedure	Average admission rate ( λ )	Average hospitalization rate (μ)	Probability of overload ( ρ ) %	Average number of users in the queue (Lq)
Sequencial procedures	597.5	599.1	100	362.3
Partial colectomy	70.9	71.7	99	93.7
Abdominal recto-sigmoidectomy	65.1	65.8	99	88.6
Treatment with multiple surgeries	63.4	64.2	99	76.9
Radical prostatovesiculectomy	93.5	95.0	98	61.6

λ : Average admission rate (hospitalized individuals/day); μ: Average hospitalization rate (hospitalized individuals/day); ρ : Probability of an overloaded system (%); Lq: Average number of users in the queue (individuals/day).
