# Supplementary material for: Bed demand and occupancy within the Brazilian National Health System for the most common types of cancer in Brazil, from 2018 to 2021: a cross-sectional study
Source: Epidemiol Serv Saude. 2024 Aug 23;33:e20231172. doi: 10.1590/S2237-96222024v33e20231172.en (PMC11348741; doi:10.1590/S2237-96222024v33e20231172.en)
Supplement: Translation

# Translation: Demanda e ocupação de leitos do Sistema Único de Saúde para os principais tipos de câncer no Brasil, de 2018 a 2021: estudo transversal

**DOI:** 10.1590/S2237-96222024v33e20231172.pt

**Authors:** Mariana Araujo Neves Lima, Daniel Antunes Maciel Villela

**Affiliations:** 1 Fundação Oswaldo Cruz, Escola Nacional de Saúde Pública Sergio Arouca, Rio de Janeiro, RJ, Brasil; 2 Fundação Oswaldo Cruz, Programa de Computação Científica da Fiocruz (Procc), Rio de Janeiro, RJ, Brasil; 3 Center for Health Wellbeing, School of Public and International Affairs, Princeton University, Princeton, Estados Unidos

**Keywords:** Acesso aos Serviços de Saúde, Equidade no Acesso aos Serviços de Saúde, Neoplasias, Pesquisa Operacional, Teoria de filas

## Abstract

**Objetivo:**

Analisar a demanda e a ocupação de leitos do Sistema Único de Saúde para os principais tipos de câncer no Brasil, de 2018 a 2021.

**Métodos:**

Estudo transversal, descritivo, com dados do Sistema de Informação Hospitalar. Por meio do modelo de teoria de filas, foram calculados: taxa média de entrada para internação, taxa média de internação, probabilidade de sobrecarga e número médio de pessoas em fila.

**Resultados:**

As regiões Sudeste e Sul apresentaram as maiores taxas médias de internação, enquanto a região Norte obteve as menores taxas. A região Sudeste obteve alta probabilidade de sobrecarga de leitos cirúrgicos, principalmente São Paulo (99,0%), Minas Gerais (97,0%) e Rio de Janeiro (97,0%). São Paulo mostrou sobrecarga acima de 95,0% em todos os tipos de leitos analisados.

**Conclusão:**

Constatou-se alta probabilidade de ocupação de leitos oncológicos no SUS, em especial os cirúrgicos e clínicos, e disparidades regionais na sobrecarga dos leitos.

## Contribuições do estudo

**Principais resultados:**

Constatou-se a elevada demanda por internações em leitos oncológicos na região Sudeste e alta probabilidade de sobrecarga nos estados das regiões Sudeste e Nordeste do Brasil, observando-se assim as iniquidades de acesso aos serviços de saúde no país.

**Implicações para os serviços:**

Implicações para os serviços

**Perspectivas:**

Este estudo apresenta uma metodologia para melhor alocação de recursos e gestão de fluxos de leitos cirúrgicos e clínicos, nos territórios com maior sobrecarga de leitos e regiões com baixa oferta de serviços.

## INTRODUÇÃO

O Brasil apresenta tendência de aumento na incidência de câncer devido ao envelhecimento populacional, mudanças nos estilos de vida e aumento das exposições ambientais e ocupacionais^.^ 1 Segundo dados mais recentes sobre a incidência de câncer no Brasil, foram estimados mais de 700 mil novos casos de câncer em 2023^.^ 1 Entre eles, os mais incidentes foram os cânceres de mama, próstata, cólon e reto, pulmão, estômago e de colo do útero^.^ 1

O Sistema Único de Saúde (SUS) é composto por uma rede regionalizada e hierarquizada que busca ofertar o atendimento integral de forma universal à população brasileira^.^ 2 A estruturação em Redes de Atenção à Saúde engloba desde a promoção da saúde, prevenção e rastreamento do câncer até os cuidados paliativos. A rede hospitalar de assistência oncológica é composta por Unidades de Assistência de Alta Complexidade em Oncologia (UNACON) e os Centros de Assistência de Alta Complexidade em Oncologia (CACON)^.^ 3

Apesar da existência de uma rede hospitalar de assistência oncológica, estudos anteriores constataram desigualdades regionais de acesso aos serviços de saúde no SUS. Tais pesquisas buscaram identificar o fluxo origem/destino das internações de pessoas com câncer de mama^,4,^ 5 câncer do aparelho digestiv^o^ 6 e para os tipos de tratamento: quimioterapia, radioterapia e cirurgia^.^ 7 As regiões Sudeste e Nordeste são polos de atendimento oncológico, ou seja, atraem maior demanda por atendimento, podendo acarretar filas nessas regiões, e vazios sanitários, sobretudo na região Norte^.4,5,^ 7

No Brasil, há déficit de leitos hospitalares gerais, em especial no SUS. Em 2017, países da Organização para a Cooperação e Desenvolvimento Econômico tinham em média 4,7 leitos hospitalares por mil pessoas, ao passo que no Brasil a média era de 2,3 leitos por mil habitantes^.^ 8 Em relação aos leitos de Unidade de Terapia Intensiva (UTI), em 2020, o país dispunha de 1,4 leito no SUS para cada 10 mil habitantes, contra 4,9 da rede privada^.^ 9 Além disso, estados como Amapá, Roraima, Acre, Maranhão, Piauí e Bahia apresentam menos de 1 leito por 10 mil habitantes, ou seja, menos do que o quantitativo recomendado pela Organização Mundial da Saúde (OMS) de 1 a 3 leitos por 10 mil habitantes^.^ 10

Apesar de não haver um número de leitos recomendados, o Ministério da Saúde propôs critérios e parâmetros para a determinação da quantidade de leitos gerais, considerando quatro componentes^.^ 11 Um estudo comparativo sobre os parâmetros de oferta entre as portarias de 2002 e 2015 constatou que a distribuição da oferta de leitos gerais e de UTI pelo território nacional não é a ideal para atender toda a população de maneira equitativa^.^ 12 A indisponibilidade de leitos para tratamento oncológico é crítica, pois usualmente os usuários têm necessidade de leitos cirúrgicos para realização de cirurgia curativa ou paliativa, leitos clínicos para internações, tratamento de intercorrências e complicações, e leitos de UTI. Em casos de insuficiência de leitos, pode haver suspensão de cirurgias eletivas, falha no fluxo de transferências entre as unidades assistenciais e uso inapropriado dos leitos, o qual também está relacionado com a gestão dos leitos^.^ 13 Dessa forma, pode haver demora para o início do tratamento. 

Diante disso, a avaliação da sobrecarga dos sistemas auxilia os gestores de saúde na tomada de decisão no planejamento da capacidade ideal de serviço de saúde, melhorias nos fluxos de atendimento para usuários com câncer e na alocação de recursos^.^ 14 Nessa perspectiva, o presente estudo utilizou o modelo de teoria de fila^s^ 15 com o objetivo de analisar a demanda e ocupação de leitos do SUS para os principais tipos de câncer no Brasil, de 2018 a 2021.

## MÉTODOS

### Desenho de estudo

Trata-se de um estudo transversal descritivo, com dados de internações hospitalares devido a condições clínicas agudas e crônicas relacionadas diretamente ao câncer, notificadas entre 2018 e 2021.

#### Contexto

A rede hospitalar para o atendimento oncológico no Brasil dispõe de 359 instituições cadastradas. Vale ressaltar que 265 hospitais são habilitados como UNACON, nas quais ocorre o atendimento dos principais tipos de cânceres mais prevalentes no país, e 44 unidades são classificadas como CACON com atendimento para todos os tipos de câncer^.^ 16 Em 2019 a Portaria GM/MS nº 1.39^9^ 17 estabeleceu a presença de um hospital habilitado a cada mil casos novos anuais de câncer estimados. 

Entre 2018 e 2021, foram realizadas 898.724 internações, sendo 224.532 em 2018, 239.603 em 2019, 220.559 em 2020 e 214.030 em 2021, resultando em média 2019,5 internações por ano. No presente estudo, foram selecionados os registros com data de internação e data de saída entre 1/1/2018 e 31/12/2021 segundo Unidade da Federação (UF). Esse período de análise foi considerado mediante a disponibilidade dos dados atualizados. Como abrangeu dois anos da pandemia de covid-19, optamos por incluir os dois anos que os antecederam.

### Participantes

A amostra do estudo abrangeu as internações cadastradas no Sistema de Informação Hospitalar (SIH/SUS), entre 1/1/2018 e 31/12/2021, com os seguintes códigos de diagnóstico principal da Classificação Estatística Internacional de Doenças e Problemas Relacionados à Saúde (CID-10): C5 (câncer de mama), C53 (câncer do colo do útero), C61 (câncer de próstata), C34 (câncer dos brônquios e dos pulmões) e os códigos C18 a C20 (câncer colorretal).

### Variáveis

- Número da Autorização de Internação Hospitalar (AIH);
- Código do diagnóstico principal (CID-10): câncer de mama (CID-10: C50), câncer do colo do útero (CID-10: C53), câncer de próstata (CID-10: C61), câncer dos brônquios e dos pulmões (CID-10: C34) e câncer colorretal (CID-10: C18 a C20)
- Data de internação;
- Data de saída (alta);
- Sexo: feminino e masculino;
- Grupo etário em anos: até 18, 19 a 39, 40 a 59, 60 a 69, 70 a 79, 80 a 89, 90 ou mais;
- Raça/cor da pele: amarela, branca, indígena, parda e preta;
- Região: Centro-Oeste, Nordeste, Norte, Sudeste, Sul;
- Ano de tratamento: 2018, 2019, 2020 e 2021;
- Especialidade do leito: cirúrgico, clínico e outros;
- Internação por complicação aguda ou crônica e tratamento de intercorrências e complicações;
- Internação por procedimentos cirúrgicos sequenciais: consiste na internação quando os procedimentos cirúrgicos ocorrem sob o mesmo ato anestésico;
- Internação para tratamento quimioterápico de administração contínua: internação para tratamento quimioterápico de administração endovenosa contínua de 24 horas.

### Medidas do modelo de teoria de filas

- Taxa média de entrada para internação (λ): corresponde ao ritmo médio de usuários que chegam para serem atendidos em leito hospitalar por dia. Essa variável representa a demanda por internação hospitalar;
- Taxa média de internação (μ): consiste no número de internações por unidade de tempo, que é a variável representativa da oferta de serviços no sistema;
- Probabilidade de sobrecarga (ρ): diz respeito ao congestionamento dos serviços. Essa medida é calculada pela equação ​​ $P(n>0)=\rho =\left(\frac{\lambda }{µ}\right)$

- Número médio de usuários na fila (Lq): em inglês, *queue length*, que representa o comprimento da fila, ou seja, a média de usuários na fila aguardando um leito hospitalar. Para o seu cálculo é utilizada a seguinte equação: Lq =  ^15^

### Fonte de dados

Foram coletados dados referentes às internações em leitos clínicos, cirúrgicos e em UTI por meio do SIH/SUS^.^ 18 Sendo assim, ao serem coletados dados de internações hospitalares no período de análise, o mesmo usuário pode ter sido computado em várias internações. Dessa forma, cada internação em leito hospitalar foi decorrente de uma fila para tal. Para a extração dos dados de forma automática, utilizou-se o pacote Microdatasus, presente na plataforma R^.^ 19

### Análise estatística

Para a análise utilizou-se a teoria de filas, na qual as UFs foram analisadas como unidades de entrada de usuários com sintomas e com capacidade de atendimento para diagnóstico e tratamento. Os componentes principais do modelo de filas incluem: taxa média de chegada (λ), taxa média de serviço (μ), número de servidores, capacidade de serviço e a disciplina da fila, ou seja, a ordem de chegada dos usuários. 

Primeiramente, realizou-se análise descritiva das características da população de estudo (sexo, faixa etária, raça/cor da pele, região, ano e especialidade). Convencionou-se a taxa média de chegada como taxa média de entrada para internação e taxa de serviço como taxa média de internação. 

Por se tratar de internações em leitos hospitalares, primeiramente calculou-se o tempo médio de permanência hospitalar (Tabela Suplementar 1). Nesse indicador, o numerador foi o somatório do número de usuários/dia no período de estudo e o denominador foi o número de saídas no período. Para realizar o cálculo de usuários/dia, construiu-se um banco de dados no qual cada entrada foi uma internação individual. 

A modelagem da taxa média de entrada para internação se deu através do processo de Poisson com distribuição exponencial, uma vez que se assume uma distribuição discreta de eventos com um grande número de usuários com entradas independentes^.^ 15 Nesse caso, os eventos são os novos registros hospitalares por unidade de tempo (dia). Este pressuposto foi avaliado de forma gráfica. 

Optou-se por utilizar o modelo M/M/1, dado que os intervalos entre as chegadas e as taxas médias de internação seguem distribuição exponencial independente e identicamente distribuída. Nesse modelo, M significa Markoviano e 1 simboliza que a fila tem apenas um único servidor atendendo os usuários por ordem de chegada. Esse modelo é caracterizado pela disciplina FIFO, do inglês *first in, first out* “primeiro a chegar, primeiro a sair”. No presente estudo, a fila é decorrente de um único servidor de leitos e o atendimento se dá por ordem de chegada, pois o usuário é encaminhado para a unidade de tratamento em questão por meio do encaminhamento no SUS.

A partir dos parâmetros de taxa média de entrada para internação (λ) e taxa média de internação (µ), pode-se calcular a probabilidade de sobrecarga (ρ) e o número médio de usuários na fila (Lq). Quando a taxa média de entrada para internação (λ) é maior do que a taxa de saída (μ), interpreta-se como o aumento da probabilidade de filas. 

### Considerações éticas

Por se tratar de dados secundários, não identificáveis, não houve necessidade de registro e aprovação da pesquisa por Comitê de Ética em Pesquisa.

## RESULTADOS

Foram selecionadas 912.567 internações para os tipos de câncer analisados entre 2018 a 2021. Após excluir dados incompletos, foram contabilizados 898.724 registros de AIHs para pessoas com câncer de mama (N = 271.861), colo do útero (N = 90.039), colorretal (N = 314.537), próstata (N = 125.519) e pulmão (N = 96.768) (Tabela 1). 

**Tabela 1:** Distribuição das internações pelos principais tipos de câncer no Brasil segundo variáveis sociodemográficas e especialidade do leito, 2018-2021

**Variáveis**	**Total N = 898.724**	**Câncer de colo do útero N = 90.039**	**Câncer colorretal N = 314.537**	**Câncer de mama N = 271.861**	**Câncer de próstata N = 125.519**	**Câncer de pulmão N = 96.768**
	**n (%)**	**n (%)**	**n (%)**	**n (%)**	**n (%)**	**n (%)**
**Sexo**						
Feminino	557.054 (62,0)	90.021 (100,0)	153.633 (49,0)	268.988 (99,0)	NA	44.412 (46,0)
Masculino	341.670 (38,0)	NA	160.904 (51,0)	2.873 (1,0)	125.519 (100,0)	52.356 (54,0)
**Faixa etária (em anos)**						
Até 18	4.532 (0,5)	105 (0,1)	2.830 (0,9)	796 (0,3)	170 (0,1)	631 (0,7)
19 a 39	82.091 (9,1)	26.235 (29,0)	21.185 (6,7,0)	31.365 (12,0)	177 (0,1)	3.129 (3,2)
40 a 59	342.546 (38,0)	43.001 (48,0)	116.281 (37,0)	137.641 (51,0)	18.525 (15,0)	27.098 (28,0)
60 a 69	251.755 (28,0)	12.536 (14,0)	94.500 (30,0)	60.843 (22,0)	48.416 (39,0)	35.460 (37,0)
70 a 79	161.479 (18,0)	6.251 (6,9)	60.379 (19,0)	30.463 (11,0)	41.165 (33,0)	23.221 (24,0)
80 a 89	51.012 (5,7)	1.738 (1,9)	17.889 (5,7)	9.631 (3,5)	15.058 (12,0)	6.696 (6,9)
90 ou mais	5.309 (0,6)	173 (0,2)	1.473 (0,5)	1.122 (0,4)	2.008 (1,6)	533 (0,6)
**Raça/cor da pele**						
Amarela	12.068 (1,5)	1.498 (1,9)	4.065 (1,4)	3.442 (1,4)	1.674 (1,6)	1.389 (1,7)
Branca	414.577 (52,0)	33.133 (43,0)	170.413 (61,0)	118.966 (49,0)	46.104 (43,0)	45.961 (56,0)
Indígena	343 (< 0,1)	135 (0,2)	85 (< 0,1)	61 (< 0,1)	28 (< 0,1)	34 (< 0,1)
Parda	314.118 (40,0)	38.413 (49,0)	93.565 (33,0)	103.272 (42,0)	49.053 (46,0)	29.815 (36,0)
Preta	50.203 (6,3)	4.775 (6,1)	12.705 (4,5)	17.385 (7,2)	10.705 (10,0)	4.633 (5,7)
**Região**						
Centro-Oeste	51.712 (5,8)	6.393 (7,1)	17.670 (5,6)	14.945 (5,5)	7.050 (5,6)	5.654 (5,8)
Nordeste	177.630 (20,0)	23.409 (26,0)	44.137 (14,0)	60.970 (22,0)	30.947 (25,0)	18.167 (19,0)
Norte	28.846 (3,2)	7.048 (7,8)	6.265 (2,0)	8.773 (3,2)	3.597 (2,9)	3.163 (3,3)
Sudeste	425.812 (47,0)	35.850 (40,0)	144.538 (46,0)	136.769 (50,0)	64.889 (52,0)	43.766 (45,0)
Sul	214.724 (24,0)	17.339 (19,0)	101.927 (32,0)	50.404 (19,0)	19.036 (15,0)	26.018 (27,0)
**Ano do tratamento**						
2018	222.479 (25,0)	21.876 (24,0)	76.289 (24,0)	67.994 (25,0)	32.394 (26,0)	23.926 (25,0)
2019	239.360 (27,0)	23.948 (27,0)	81.506 (26,0)	73.093 (27,0)	34.714 (28,0)	26.099 (27,0)
2020	221.196 (25,0)	22.438 (25,0)	78.813 (25,0)	65.884 (24,0)	29.582 (24,0)	24.479 (25,0)
2021	215.689 (24,0)	21.777 (24,0)	77.929 (25,0)	64.890 (24,0)	28.829 (23,0)	22.264 (23,0)
**Especialidades**						
Cirúrgicos	426.081 (47,0)	39.088 (43,0)	118.919 (38,0)	175.940 (65,0)	71.630 (57,0)	20.504 (21,0)
Clínicos	449.465 (50,0)	47.310 (53,0)	189.781 (60,0)	89.771 (33,0)	50.167 (40,0)	72.436 (75,0)
Outros	23.178 (2,6)	3.641 (4,0)	5.837 (1,9)	6.150 (2,3)	3.722 (3,0)	3.828 (4,0)

NA: Não se aplica.

A população de estudo foi composta majoritariamente por indivíduos do sexo feminino (62,0%) e com idade entre 40 e 69 anos (66,0%). Entre as internações por câncer do colo do útero, a faixa etária com maior frequência foi entre 40 e 59 anos (48,0%). Aproximadamente 50% das internações do sexo feminino por câncer de mama tinham idade entre 40 e 59 anos, enquanto as internações por câncer de próstata, 84,0% dos casos, tinham idade entre 60 e 89 anos. Essa faixa etária também foi predominante em casos de câncer de pulmão (68,0%). Em internações de câncer colorretal predominou a faixa etária de 40 a 59 anos (37,0%) (Tabela 1).

A distribuição de internações por câncer colorretal foi similar entre os sexos, e 54% das internações por câncer de pulmão foram do sexo masculino. A maioria dos registros era de raça/cor branca (52,0%). A região Sudeste apresentou 47,0% das internações. Para o câncer do colo do útero, a região Sudeste correspondeu a 40,0% das internações e a região Nordeste apresentou 26,0% das internações. Para o câncer colorretal, as regiões com maior percentual de internações foram Sudeste (46,0%) e Sul (32,0%). A região Sudeste obteve 50,0% das internações por câncer de mama e 52,0% para câncer de próstata. Para o câncer de pulmão, 45,0% das internações foram da região Sudeste e 27,0% da região Sul. Em 2019, observou-se o maior quantitativo de internações (239.360 internações). As internações em leito clínico foram mais frequentes em pessoas com câncer colorretal (60,0%) e pulmão (75,0%); já para câncer de mama (65,0%) e próstata (57,0%), foram mais recorrentes em leitos cirúrgicos. Todos as variáveis foram estatisticamente significantes (Tabela 1).

As regiões Sudeste e Sul obtiveram elevada taxa média de internação para cirurgia, em especial os seguintes estados: São Paulo (525,0), Minas Gerais (269,9), Paraná (173,1) e Rio de Janeiro (169,9). Em contrapartida, a região Norte apresentou os menores valores de taxa média de internação, nos seguintes estados: Acre (11,3), Amapá (12,0), Tocantins (15,7) e Roraima (16,4). A probabilidade de sobrecarga foi igualmente elevada na região Sudeste, sendo 99,0% em São Paulo e 97,0% em Minas Gerais e Rio de Janeiro. Um total de 383 pessoas esperaram na fila por um leito cirúrgico a cada dia no estado de São Paulo (Tabela 2). 

**Tabela 2:** Medidas de desempenho de filas para internações pelos principais tipos de câncer no Brasil segundo leito clínico, cirúrgico e de UTI, 2018-2021

**UF**	**Leito clínico**				**Leito cirúrgico**				**Leito de UTI**			
	**Taxa média de entrada para internação (**λ**)**	**Taxa média de internação ( μ )**	**Probabilidade de sobrecarga (**ρ**) %**	**Número médio de usuários na fila (Lq)**	**Taxa média de entrada para internação (**λ**)**	**Taxa média de internação ( μ )**	**Probabilidade de sobrecarga (**ρ**) %**	**Número médio de usuários na fila (Lq)**	**Taxa média de entrada para internação (**λ**)**	**Taxa média de internação ( μ )**	**Probabilidade de sobrecarga (**ρ**) %**	**Número médio de usuários na fila (Lq)**
São Paulo	473,1	478,2	99,0	437,1	517,8	525,0	99,0	383,5	104,3	110,0	95,0	93,9
Minas Gerais	269,1	274,2	98,0	234,1	261,2	269,9	97,0	154,9	54,2	60,6	89,0	40,2
Rio Grande do Sul	195,0	199,6	98,0	191,0	158,6	165,9	96,0	131,3	22,8	28,3	84,0	20,3
Paraná	286,8	294,6	97,0	155,3	163,9	173,1	95,0	92,0	34,9	43,4	80,0	17,7
Rio de Janeiro	153,8	158,1	97,0	171,5	164,1	169,9	97,0	149,4	33,2	37,7	88,0	38,0
Santa Catarina	119,0	124,4	95,0	91,9	91,7	100,3	93,0	58,1	14,5	20,9	74,0	8,8
Pernambuco	116,4	121,8	95,0	98,6	92,4	100,0	92,0	52,5	15,9	21,6	73,0	10,5
Bahia	79,0	82,9	95,0	96,6	115,9	124,0	93,0	65,7	17,2	22,6	76,0	13,0
Espírito Santo	75,3	81,2	92,0	53,3	56,0	64,8	87,0	28,7	12,3	19,3	66,0	6,1
Maranhão	36,6	40,9	90,0	40,0	39,2	46,6	85,0	23,2	12,2	17,5	71,0	8,5
Ceará	32,3	36,2	90,0	40,3	72,4	79,8	91,0	43,8	10,5	16,4	69,0	8,3
Distrito Federal	31,7	35,5	89,0	37,2	28,4	34,6	83,0	19,6	6,5	10,5	64,0	5,3
Pará	21,1	24,3	87,0	29,5	23,6	27,6	85,0	23,1	8,5	12,1	71,0	9,2
Rio Grande do Norte	43,9	50,1	86,0	26,0	39,0	48,4	82,0	17,9	9,5	17,3	56,0	3,7
Alagoas	36,6	42,1	86,0	26,1	21,9	30,3	74,0	10,0	6,9	13,2	52,0	2,9
Goiás	35,5	41,6	85,0	24,4	72,4	79,8	91,0	43,8	9,1	16,8	61,0	4,3
Paraíba	24,1	29,2	82,0	19,3	32,1	39,5	81,0	16,6	8,8	15,0	60,0	4,9
Mato Grosso do Sul	30,6	37,2	81,0	17,6	22,8	30,2	78,0	12,9	6,8	13,7	51,0	2,6
Piauí	17,9	22,1	81,0	16,8	27,1	35,0	78,0	13,0	6,9	12,9	54,0	3,6
Sergipe	9,3	11,5	81,0	17,0	12,8	19,9	69,0	7,0	5,8	9,6	63,0	5,5
Mato Grosso	25,2	31,1	80,0	15,5	23,5	30,9	78,0	12,7	8,0	14,7	58,0	3,7
Amazonas	11,3	14,9	78,0	12,7	17,8	23,7	77,0	11,7	6,3	11,1	59,0	4,1
Rondônia	18,3	24,3	75,0	11,5	14,4	22,0	68,0	6,6	7,2	14,6	51,0	2,7
Tocantins	14,8	19,6	75,0	11,6	10,2	15,7	69,0	7,4	5,5	10,4	53,0	3,2
Amapá	6,5	9,2	72,0	9,1	6,4	12,0	60,0	4,0	5,1	8,5	60,0	5,2
Acre	6,9	10,7	65,0	6,0	6,2	11,3	58,0	4,6	5,4	9,6	57,0	4,0
Roraima	7,5	12,0	64,0	5,4	6,4	16,4	43,0	1,6	5,1	10,3	50,0	2,7

UTI: Unidade de Terapia Intensiva; λ: Taxa média de entrada para internação (indivíduos internados/dia); μ: Taxa média de internação (indivíduos internados/dia); ρ: Probabilidade de sistema sobrecarregado (%); Lq: Número médio de usuários na fila (indivíduos/dia).

O estudo encontrou que a média diária de internações em leitos clínicos no estado de São Paulo foi de 473,1 pessoas por dia e 437,1 pessoas aguardaram para serem atendidos por dia no estado, totalizando em 99,0% a probabilidade de encontrar o sistema sobrecarregado. Entre os estados com mais de 95,0% de probabilidade de sobrecarga para leitos clínicos, encontraram-se três da região Sudeste, três da região Sul e dois da região Nordeste, Bahia e Pernambuco (Tabela 2).

Foram analisados 75.807 leitos em UTI. Em comparação aos demais tipos de leito analisados, leitos em UTI encontravam-se menos sobrecarregados e, assim, com menor número de usuários na fila. Apesar disso, São Paulo apresentou 95% de probabilidade de leitos em UTI ocupados. As regiões Norte e Nordeste apresentaram baixa taxa de chegada para leitos em UTI (Tabela 2).

As Figuras 1 e 2 mostram que houve maior taxa média de admissão hospitalar e elevada taxa média de internação em leitos cirúrgicos para os tipos de câncer de mama e colorretal. O estado de São Paulo obteve as maiores taxas de entrada para internação, assim como as maiores taxas média de internação para todos os tipos de câncer analisados. As internações por câncer de cólon e reto tiveram maior probabilidade de sobrecarga da rede assistencial, tendo os estados de São Paulo, Rio de Janeiro, Minas Gerais e Rio Grande do Sul apresentado uma probabilidade de sobrecarga de leitos cirúrgicos de 99,0% para esse tipo de câncer. A probabilidade de sobrecarga de demanda para leitos cirúrgicos para os casos de câncer de mama também foi elevada, principalmente nos estados de São Paulo (99,0%), Rio de Janeiro (97,0%) e Minas Gerais (97,0%) (Figura 3). Os procedimentos cirúrgicos apresentaram alta probabilidade de ocupação de leitos e número elevado de usuários em fila (Tabela Suplementar 2, Figura Suplementar 1).

**Figura 1:** Taxa média de entrada para internação em leitos cirúrgicos segundo Unidade da Federação e diagnóstico, 2018-2021

**Figura 2:** Taxa média de internação em leitos cirúrgicos segundo Unidade da Federação e diagnóstico, 2018-2021

**Figura 3:** Probabilidade de sistema ocupado para leitos cirúrgicos segundo Unidade da Federação e diagnóstico, 2018-2021

## DISCUSSÃO

Os resultados do estudo corroboram os resultados de outros estudos que constataram disparidades regionais na oferta de leitos oncológicos na rede pública no Brasil^.4,5,^ 7 Os estados das regiões Sudeste e Sul foram os que apresentaram as taxas médias de internação (µ) mais elevadas em comparação com a região Norte, onde se observaram as menores taxas de internação para os leitos cirúrgicos, clínicos e UTI. Em relação aos tipos de câncer, os de cólon e reto tiveram maior probabilidade de sobrecarga de leitos cirúrgicos, clínicos e UTI, destacando-se a estimativa de que 654 usuários com câncer de cólon e reto foram estimados em fila para leitos cirúrgicos. Entre os procedimentos analisados, as cirurgias colectomia parcial e retossigmoidectomia abdominal obtiveram 99% de probabilidade de sobrecarga de leitos.

O déficit de leitos hospitalares no SUS foi igualmente observado em estudo que encontrou uma taxa de 1,6 leito SUS/mil habitantes na região Norte e de 5,0 e 5,2 leitos privados/mil habitantes nas regiões Norte e Centro-oeste, respectivamente^.^ 20 Os achados do presente estudo corroboram essas estimativas, como se pode observar em relação às taxas encontradas nos estados da região Norte, que apresentaram as menores taxas média de internação, evidenciando a baixa oferta de leitos na região, o que demanda ações visando à redução dessas iniquidades.

A região Norte foi considerada a região mais crítica em estudo prévio^,^ 21 pois dispõe de apenas 12 estabelecimentos credenciados para tratamento oncológico, incluindo-se 11 serviços de câncer de alta complexidade. Em consequência, usuários das regiões Norte e Centro-Oeste apresentaram maior deslocamento para unidade de tratamento; contudo, as regiões Sudeste e Nordeste são polos de atração, sendo Barretos o principal polo de atração no Brasil^.5,^ 7 No presente estudo, pode-se perceber que a baixa taxa de entrada no sistema da região Norte sugere a busca dos usuários por atendimento em outras regiões, que seria evidenciada pela baixa sobrecarga de leitos na região e maior sobrecarga em estados das regiões Nordeste e Sudeste.

A formação de filas ocorre se a demanda de usuários ultrapassa a capacidade do sistema de fornecer serviços em certo tempo^.^ 22 Consequentemente, atrasos na oferta de leitos podem acarretar cancelamento de cirurgias eletivas, atraso em procedimentos clínicos e em emergência, alocação de leitos inapropriados, sem se considerar sexo e tipo do leito^.^ 23 Dessa forma, os déficits de leitos podem afetar a sobrevida do paciente, a qualidade de vida e a mortalidade relacionada ao câncer. 

Um estudo de base populacional estimou a demanda global por cirurgia oncológica em 183 países entre 2018 e 2040 e encontrou que o número de casos de câncer com indicação cirúrgica aumentará 52% nesse período, totalizando 5 milhões de procedimentos. Em países de renda média-alta e média-baixa, ocorrerá o maior número absoluto de incremento de casos de câncer com tratamento cirúrgico^.^ 24 Com o aumento da demanda por cirurgia de câncer, faz-se necessário o planejamento adequado de força de trabalho e insumos para a prestação de serviços cirúrgicos.

Apesar de não ter sido o enfoque do trabalho, a pandemia de covid-19 iniciada em 2020 pode influenciar nos achados do estudo. A necessidade do isolamento social pode impactar em redução da taxa média de entrada para internação, assim como a disponibilidade de leitos pode influenciar na diminuição da taxa média de internação. Estudos anteriores encontraram queda nos dias de internação e redução no volume de cirurgias^.25,^ 26

O modelo de fila M/M/1 utilizado foi compatível com as necessidades do estudo, pois esse modelo é composto de uma fila atendida por apenas um servidor por ordem de chegada. Apesar de haver várias unidades de serviço, a regulação de leitos pelo SUS forma uma fila única, sendo, portanto, considerado apenas um servidor. Além disso, para as cirurgias eletivas oncológicas, a fila de leitos cirúrgicos é atendida por ordem de chegada ao hospital, segundo a marcação da cirurgia, e ela se dá por apenas um servidor.

O trabalho levou em consideração o tempo médio hospitalar para os cálculos das medidas de desempenho do sistema. Dessa forma, englobou as diferenças clínicas dos tipos de câncer analisados que podem influir na sobrecarga do sistema, como foi possível observar nas diferenças entre o câncer de cólon e reto e o câncer de próstata. Este último tem menor tempo médio de internação hospitalar e obteve baixa sobrecarga de leitos. 

Este trabalho apresenta limitações. A base de dados do SIH não abrange usuários da rede de saúde suplementar, sendo assim, não foi possível diferenciar a probabilidade de sobrecarga entre leitos do SUS e particulares. O SIH não apresenta procedimentos segundo a gravidade clínica, nem constam variáveis clínicas, como o estadiamento do câncer, fatores esses que podem influenciar na entrada e saída do sistema de fila^.^ 18 O banco de dados dispõe a entrada dos usuários do SUS por internações, logo não permite individualizar a amostra selecionada, podendo, assim, introduzir um viés, dado que o mesmo paciente pode ser incluído mais de uma vez no estudo. No entanto, espera-se que tais casos representem uma pequena parcela da amostra estudada. 

Uma limitação dos dados utilizados diz respeito ao possível viés de acesso ao diagnóstico. O estado de São Paulo apresentou as maiores taxas de entrada e de internações, mas tem população numerosa e alto número de diagnósticos, o que consequentemente aumenta a taxa média de entrada para internações. Entretanto, em estados com limitações para realizar o diagnóstico podem ter mais casos do que realmente são reportados, mas a medida de entrada para internação será menor. Em relação ao modelo M/M/1 escolhido, tem como limitação a falta de incorporação de variáveis demográficas, como o tamanho ou a distância média para o centro hospitalar, que podem impactar na busca até a unidade de referência e, consequentemente, na fila por leitos hospitalares. Além disso, vale ressaltar que os resultados estaticamente significantes da análise descritiva sobre as variáveis sociodemográficas e a especialidade no leito podem ser influenciados pelo grande número de observações.

Conclui-se que há evidências de desigualdades regionais no acesso a internações clínicas, cirúrgicas e em leitos de UTI no Brasil. O estudo observou déficit de leitos entre usuários do SUS para tratamento oncológico na região Norte e alta probabilidade de sobrecarga nas regiões Sudeste e Nordeste do país. Além disso, o estudo mostrou que principalmente as pessoas com câncer de cólon e reto têm alta probabilidade de enfrentar filas para internação.

## Tabela Suplementar 1

**Table:** Tempo médio de permanência (em dias) em leito hospitalar por câncer de mama, próstata, colorretal, colo do útero e pulmão no Brasil, 2018-2021

Variáveis	Leitos em clínica médica	Leitos cirúrgicos	Leitos de UTI
Região			
Norte	8,8	6,4	12,0
Sudeste	6,6	5,2	10,5
Nordeste	7,3	4,4	9,7
Centro-Oeste	6,3	4,8	10,7
Sul	5,3	5,0	10,0
Diagnóstico			
Câncer de pulmão	8,3	7,2	10,1
Câncer de colo do útero	8,2	4,5	12,0
Câncer colorretal	4,8	8,3	11,0
Câncer de próstata	7,4	4,5	8,3
Câncer de mama	6,9	2,8	8,9
Especialidade			
Clínicos	6,4	-	12,2
Cirúrgico	-	5,0	9,7
Ano			
2018	6,7	5,2	10,9
2019	6,7	5,1	10,6
2020	6,2	4,9	10,1
2021	6,0	4,7	9,7

UTI: Unidade de Terapia Intensiva.

## Tabela Suplementar 2

**Table:** Medidas de desempenho de filas para internações em leito cirúrgico segundo procedimento, 2018-2021

Procedimento	Taxa média de entrada para internação (λ)	Taxa média de internação (μ)	Probabilidade de sobrecarga (ρ) %	Número médio de usuários na fila (Lq)
Procedimentos sequenciais	597,5	599,1	100	362,3
Colectomia parcial	70,9	71,7	99	93,7
Retossigmoidectomia abdominal	65,1	65,8	99	88,6
Tratamento com cirurgias múltiplas	63,4	64,2	99	76,9
Prostatovesiculectomia radical	93,5	95,0	98	61,6

λ: Taxa média de entrada para internação (indivíduos internados/dia); μ: Taxa média de internação (indivíduos internados/dia);ρ: Probabilidade de sistema sobrecarregado (%); Lq: Número médio de usuários na fila (indivíduos/dia).
